# Use of Botox Injection As a Bridge In Pediatric Recurrent Shoulder Dislocations

**Published:** 2013-10-11

**Authors:** Thomas I Lemon

**Affiliations:** School of Medicine Cardiff University

**Dear Sir,**

Recurrent gleno-humeral dislocation is not a limb threatening condition but results in great distress in adolescent patients.[1] This may potentially lead to psychiatric and long term physical disability. Due to the rarity of this condition in children, the management is not always known. Herein, we present a perhaps unorthodox but successful management of this condition as measured by reduced intensity of pain and decrease in frequency of recurrent gleno-humeral dislocations before definitive treatment could be offered.

A 16-year-old boy, who had a bicycle accident 4 years ago, suffered on-going recurrent dislocations of his left shoulder. The shoulder used to dislocate more than 10 times a day, and resulted in regular visits to Accident and Emergency Department. Previously the shoulder was normal. Aetiology of the recurrent dislocations was unclear. Imaging (Plain film X-ray and later Magnetic Resonance Imaging) of the shoulder was unremarkable. The dislocations affected greatly his daily chores. On examination, the patient was able to dislocate shoulder voluntarily in clinic – showing loss of normal shoulder contour. There was no noticeable inflammation or muscle wasting. Whilst dislocated, the pectoralis major was overtly in spasm. On palpitation, the sterno-clavicular and acromio-clavicular joints were found normal bilaterally. The acromion and coracoid processes, scapula, spine, and biceps tendon in the biciptal groove were also normal on both the sides. No tenderness of the supraspinatus tendon was present. All movements were noticeably reduced when shoulder dislocated. Patient was given inj. botox to the pectoralis major. A reduction in the severity of pain and frequency of dislocations with the first injection was noted afterwards. He is currently on repeated inj. botox therapy.

There are three types of shoulder dislocations. Anterior dislocation (95%) which is commonly sub-coracoid; it has potential to damage axillary artery. Posterior dislocation may be caused by seizures or strength imbalance of rotator cuff muscles; it is often undetected. Inferior dislocation (0.8%) also called luxation erecta.[1-5] Our case does not fit into any of these types. The pectoralis major seems to be in spasm when dislocation occurs, and the inj. botox ceases this. The plan to give repeated inj. botox to the lower part of pectoralis major lessened the severity of pain and frequency of dislocations. This has dramatically improved his daily activities. Inj. botox administration could be considered in recurrent post traumatic gleno-humeral dislocations awaiting surgical interventions, other skeletal muscle spasm (particularly oculomotor and facial spasm) and pain control (ranging from chronic pain to post haemorrhoidectomy pain).[2-5] It is important to note that long term psychiatric problems can occur in paediatric patients owing to long standing pain; inj. botox proved to be a temporary way-forward in our patient until a definite diagnosis and treatment are instituted.

## Footnotes

**Source of Support:** Nil

**Conflict of Interest:** None declared

